# Bacteriophage Procurement for Therapeutic Purposes

**DOI:** 10.3389/fmicb.2016.01177

**Published:** 2016-08-12

**Authors:** Beata Weber-Dąbrowska, Ewa Jończyk-Matysiak, Maciej Żaczek, Małgorzata Łobocka, Marzanna Łusiak-Szelachowska, Andrzej Górski

**Affiliations:** ^1^Bacteriophage Laboratory, Ludwik Hirszfeld Institute of Immunology and Experimental Therapy, Polish Academy of SciencesWroclaw, Poland; ^2^Phage Therapy Unit, Ludwik Hirszfeld Institute of Immunology and Experimental Therapy, Polish Academy of SciencesWroclaw, Poland; ^3^Institute of Biochemistry and Biophysics, Polish Academy of SciencesWarsaw, Poland; ^4^Autonomous Department of Microbial Biology, Faculty of Agriculture and Biology, Warsaw University of Life SciencesWarsaw, Poland; ^5^Department of Clinical Immunology, Transplantation Institute, Medical University of WarsawWarsaw, Poland

**Keywords:** bacteriophage isolation, therapeutic phages, experimental phage therapy, treatment of bacterial infections, antibiotic resistance

## Abstract

Bacteriophages (phages), discovered 100 years ago, are able to infect and destroy only bacterial cells. In the current crisis of antibiotic efficacy, phage therapy is considered as a supplementary or even alternative therapeutic approach. Evolution of multidrug-resistant and pandrug-resistant bacterial strains poses a real threat, so it is extremely important to have the possibility to isolate new phages for therapeutic purposes. Our phage laboratory and therapy center has extensive experience with phage isolation, characterization, and therapeutic application. In this article we present current progress in bacteriophages isolation and use for therapeutic purposes, our experience in this field and its practical implications for phage therapy. We attempt to summarize the state of the art: properties of phages, the methods for their isolation, criteria of phage selection for therapeutic purposes and limitations of their use. Perspectives for the use of genetically engineered phages to specifically target bacterial virulence-associated genes are also briefly presented.

## Introduction

The post-antibiotic era in which minor injuries and common infections can kill – because of lack of drugs or their ineffectiveness – nowadays is not an apocalyptic fantasy, but a real 21st century possible threat (WHO, 2014). For example, ESKAPE organisms (*Enterococcus faecium, Staphylococcus aureus, Klebsiella pneumoniae, Acinetobacter baumannii, Pseudomonas aeruginosa*, and *Enterobacter* species) are extremely resistant to multiple antimicrobial agents (Moellering, 2010) and are a serious challenge in medicine today.

According to the National Institutes of Health (NIH, USA), phages are innovative components that may be used to combat microbial resistance (NIH, 2014). Clinical application of phage preparations is associated with well-described and -characterized processing, and its influence on phage stability during preparation and under storage conditions should be verified and described in detail, especially as it is needed for phase III clinical trials (Vandenheuvel et al., 2015). As suggested, personalized phage therapy is probably more effective than *prêt-à-porter* preparations, as it is based on tailored preparations which can be adjusted to changing bacterial pathogens (Keen, 2012; Mattila et al., 2015).

## State of the Art

Bacteriophages are viruses which have the ability to multiply only in bacterial cells, and they are detectable almost everywhere where live bacteria exist (they have been isolated from all environments and ecological niches, e.g., inland waters, hot springs, salt water, soil, cold water) (Lin et al., 2010; Zheng et al., 2013; Zhan et al., 2015). It is estimated that in the biosphere there are approximately 10^30-31^ phages (Hendrix et al., 1999; Hendrix, 2002), which is 10-fold higher than the total number of bacterial cells (Abedon et al., 2011a). They are an inherent element of the human microbiome, and therefore they are well tolerated when used in phage therapy (Międzybrodzki et al., 2012; Reyes et al., 2012).

A serious medical and social problem results from the increasing antibiotic resistance of bacterial strains (Arias and Murray, 2009; Magiorakos et al., 2012; Deris et al., 2013; WHO, 2014). The pharmaceutical industry is withdrawing from research and development on new antibiotics due to unprofitability of the venture and the risks of development of resistance in bacteria (Clarke, 2003).

Initially, bacteriophages were isolated from stool samples of patients suffering from diarrhea and were first successfully used in the treatment of bacterial dysentery (D’Hérelle, 1917). Hankin was the first to observe that the water of the Ganga and the Yamuna rivers (India) had antiseptic properties; in particular, water samples from them were able to destroy *Vibrio cholerae* (Hankin, 1896; Abedon et al., 2011b).

In humans phages have been successfully used in the treatment of a wide range of infections, both local and systemic (Weber-Dąbrowska et al., 2001, 2003; Borysowski and Górski, 2008; Międzybrodzki et al., 2012; Borysowski et al., 2014; Rose et al., 2014). This is due to the fact that they have many features which give them advantages over antibiotics, e.g., phages multiplying at the site of infection where there are bacteria sensitive to them (Abedon and Thomas-Abedon, 2010; Loc-Carrillo and Abedon, 2011; Kutter et al., 2012). Interestingly, development of the resistance of bacteria to antibiotics does not parallel the development of phage resistance in bacteria, although the acquisition of resistance to phages by bacteria may pose a significant problem (Loc-Carrillo and Abedon, 2011). Therefore, as mentioned, phages can be used to treat infections caused by antibiotic-resistant bacterial strains (Górski et al., 2007; Kutter et al., 2010), including bacteria resistant to multiple antibiotics (Górski et al., 2007; Hanlon, 2007). Moreover, it has been demonstrated that phage therapy is safe and relatively free of side effects (Borysowski and Górski, 2008; Łusiak-Szelachowska et al., 2014; Jończyk-Matysiak et al., 2015).

The use of bacteriophages in antimicrobial phage therapy is based on their ability to recognize, adsorb and multiply only within the bacterial cell and cause its lysis (Burrowes et al., 2011). Phage therapy is defined as the use of bacteria-specific viruses to combat pathogenic bacteria that cause infections (Loc-Carrillo and Abedon, 2011). Bacteriophages are usually highly specific and may be active against antibiotic resistant Gram-positive and Gram-negative bacteria (Biswas et al., 2002; Burrowes et al., 2011; Vinodkumar Srinivasa et al., 2011; Chhibber et al., 2013).

Phages have been used as a therapeutic agent in two main centers (Weber-Dąbrowska et al., 2000a,b; Sulakvelidze et al., 2001; Clark and March, 2006): in Poland [the Ludwik Hirszfeld Institute of Immunology and Experimental Therapy, Polish Academy of Sciences in Wroclaw (IIET PAS)] and at the Eliava Institute in Tbilisi, Georgia. In 2005 the Phage Therapy Unit was opened there – the first of its type in the European Union. Over the past few years, phage therapy has gained wider interest (Denou et al., 2009; Górski et al., 2009, 2012; Międzybrodzki et al., 2012; Pirnay et al., 2012; Henein, 2013; McCallin et al., 2013; Borysowski et al., 2014; Matsuzaki et al., 2014). Moreover phages are used as therapeutics also, e.g., in former USSR and the Russian Federation (Krylov et al., 2012; Krylov, 2014; Sarker et al., 2016).

## Sources of Phage Isolation and Phage Propagation Hosts

The environment is a unique source of all types of phages, offering the possibility to isolate them for therapeutic purposes (Naghavi et al., 2013; Shukla et al., 2014). Phages are unevenly distributed, with the prime source of phages against human bacterial pathogens being the immediate hospital vicinity (Latz et al., 2016). For quality controlled production, phages active against *P. aeruginosa* and *S. aureus* were isolated from sewage and river water (Merabishvili et al., 2009). Already Dubos et al. (1943) isolated phages against *Shigella dysenteriae* 2308 from New York city sewage. The isolation of phages from environmental sources is more often possible when the sample contains the target bacterium (Gill and Young, 2011; Periasamy and Sundaram, 2013). An optimal source for searching for new phages is sewage (Łobocka et al., 2014; Weber-Dąbrowska et al., 2014). Vandersteegen et al. (2013) isolated raw inlet sewage water phages, Romulus and Remus, active against *S. aureus*. These phages infected 80% of tested strains and had the potential to destroy biofilm. It is also possible to obtain new virulent phages from stream water (Ul Haq et al., 2011). Sheep and cattle fecal samples were also a source of newly isolated phages.

The occurrence of phages against bacteria that are not abundant, in samples obtained from the environment, is usually low, so the simplest and most direct methods for their isolation may be unreliable. Therefore it is recommended to use large volume sample because concentration and/or enrichment methods may be relevant (Gill and Young, 2011). In larger sample volumes the possibility to detect phage is increased, and this type of searching may be recommended when too few desired phages are present in the sample (Gill and Hyman, 2010). Sometimes phage inactivation may occur during the waste water treatment process; e.g., during anaerobic-aerobic adsorption on solid particles as well as aerobic treatment using activated sludge or flocculants (Tanji et al., 2002). But, in Bacteriophage Laboratory IITD PAS phages active against *Enterococcus* and *Staphylococcus* strains were isolated from drinking water (Weber-Dąbrowska et al., 2014).

The broad host range phage isolation protocol from sewage and pond water was described by Jensen et al. (1998). The widely considered narrow host range of isolated phages may be the result of the isolation procedure rather than the naturally occurring phage spectrum. Host specificity of phages that are isolated from aquatic samples is broader than that of phages isolated from other types of samples (Wommack and Colwell, 2000). Two staphylococcal phages, PhiSA039 and PhiSA012, isolated from a municipal wastewater treatment plant in Tokyo were shown to have a wide host range (Synnott et al., 2009). Similarly, the P-27/HP bacteriophage isolated from sewage was polyvalent and was able to control multidrug resistant *S. aureus* (Gupta and Prasad, 2011). It was able to kill 60% of isolates of *S. aureus* from humans. Parra and Robeson (2016) found three polyvalent bacteriophages (FC, FP, and FQ) infecting the *S. enterica* serovar Choleraesuis and *Escherichia coli* strains, in wastewater samples in Chile. Yu et al. (2015) suggested that polyvalent phages may be rapidly isolated from the environment by using different sequential hosts. Their novel method is based on two sequential multihost isolations, and both culture-dependent and culture-independent phage libraries were tested for phages of broad range of infectivity. These methods were found to be useful for isolation of broadly polyvalent phages that were able to infect bacteria of distantly related taxons. Another example of unexpectedly broad host-range phage is phage JHP, which was isolated recently by Khawaja et al. (2016). Unfortunately, it is difficult to obtain phages whose specificity is broad enough to cover all strains within a pathogenic species, despite the fact that it is possible to isolate phages with a broad host range (Nilsson, 2014). A typical problem in phage therapy is a narrow host-range of certain phages. T4 phages tested by Denou et al. (2009) for the efficacy in curing *E. coli* diarrhea proved to be not only species- but also strain-specific. Therefore, it was necessary to use phage cocktails containing 10 and 16 phages that cover half to two thirds of *E. coli* strains (representing the five main pathotypes isolated from patients suffering from diarrhea). Ready-made, broad-host-range cocktails that contain phages active against different pathogens may be a good solution when rapid phage application is needed.

The richest source of the most effective therapeutic phages is the material from patients who recovered from an infection (D’Herelle, 1938). Mattila et al. (2015) isolated phages on demand from sewage. They emphasized the ease of isolation of phages against such pathogenic strains as *Pseudomonas aeruginosa, Salmonella*, extended spectra beta-lactamase *E. coli*, and *K. pneumonia*, but greater difficulty to find phages active against vancomycin-resistant *Enterococcus* and *Acinetobacter baumannii* strains. Isolation of phages that are active against *S. aureus* turned out to be the most difficult. However, e.g., Kwiatek et al. (2012) and Sahin et al. (2013) obtained phages active against *S. aureus* clinical MRSA strains.

O’Flynn et al. (2004) isolated phages active against the *E. coli* O157:H7 strain from human fecal samples of patients with gastrointestinal disorders, and from bovine feces. Certain phages were isolated from human skin swabs, in particular from bacterial strains (Brown et al., 2016), e.g., phages specific to *Propionibacterium acnes*. Raw hospital sewage samples were the source of the phiAxp-3 lytic phage which was active against *Achromobacter xylosoxidans* A22732 (Ma et al., 2016). This phage was active against four A. *xylosoxidans* strains tested.

Sometimes, it is possible to isolate a phage against certain strain of pathogenic species and then adapt this phage to another host strain against which one cannot easily isolate new phages.

Typically, high titer lysates containing phages that can effectively infect certain strains of bacterial pathogens can be obtained by the propagation of these phages in cells of related strains. Sometimes, to avoid the risk of infection and to simplify the culture procedure, a non-pathogenic bacterial strain – called a surrogate strain – may be chosen for phage isolation and propagation (Gill and Hyman, 2010). For example, David et al. (1980) chose for their research *Mycobacterium smegmatis* – a species safer than the closely related pathogenic *M. tuberculosis* – to propagate the D29 phage. This strain grew rapidly compared to the pathogenic *M. tuberculosis* and was also susceptible to the D29 phage; therefore for practical reasons it was chosen for the propagation of this phage.

## Synergy Between Phages

Some phage preparations are in the form of a cocktail – they may be composed of more than one phage. Phages in the cocktail may be active against various strains of the same bacterial species, but the killing of target bacteria by the cocktail may be more effective than expected based on the lytic activities of single cocktail phages. This phenomenon is referred to as synergy, and it may be helpful and useful from the therapeutic point of view. According to research conducted by Schmerer et al. (2014), it is possible to obtain such synergy if one phage can facilitate the infection of the same bacterium by another phage. They isolated the phage from sewage and observed that it caused the formation of large plaques on a mucoid *E. coli* strain. The large size of plaques resulted from the combined activity of two phages – J8-65 (turbid plaques with a halo effect) and T7 (forming small plaques). The combination of these two phages resulted in 10–100-fold higher killing efficacy of host bacteria compared to each phage alone. The likely cause of this synergy was the activity of J8–65 colanidase - an enzyme which degrades the exopolysaccharide of mucoid bacterial surface. By degrading a mucoid barrier protecting cells from the T7 adsorption, the colanidase provided easier access of T7 to the bacterial cell surface receptors. This observed synergy was temperature- and media-dependent. From a practical point of view, phage-encoded depolymerase enzymes may facilitate phage access to bacteria in a biofilm community (Cornelissen et al., 2011; Pires et al., 2016), which may have practical implications in biofilm treatment. Phage efficacy in destroying biofilm was observed in the case of biofilms formed by, e.g., *Enterococcus* or *Staphylococcus* (Gutiérrez et al., 2015; Khalifa et al., 2015).

The knowledge based on the possibility to obtain synergy may significantly improve production of phage preparations intended for phage therapy, as it strengthens their potential efficiency.

## Methods of Phage Isolation

Samples that are believed to contain phages may be directly – after sterilization by filtration (0.22 μm) or after incubation (enrichment method) – plated on a bacterial host lawn to search for plaques – the simplest method for visualization of phage occurrence. It is worth considering that for each phage and bacterium specific medium, growth and storage conditions should be adjusted and evaluated (Fortier and Moineau, 2009). Moreover, for new phage isolation it is recommended to use one bacterium in the exponential phase of growth (Wommack and Colwell, 2000; Kutter, 2009). To verify the findings, each test should be performed at least in duplicate.

**FIGURE 1:**
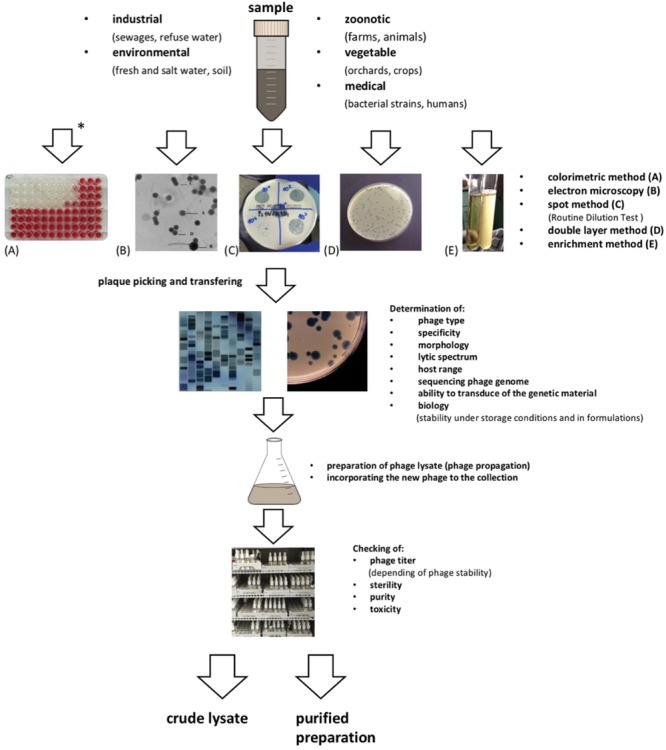
**Steps in phage isolation for therapeutic purposes**.

Liquid samples seem to be easier for phage searching, whereas solid ones need to be suspended in a sterile liquid medium. Sometimes it is possible to use pooled samples (e.g., in searching for phages in the urine of an infected patient, when the material was collected at different time points). However, independent testing seems to be a more likely method of isolating phages than pooling (Gill and Hyman, 2010). The main steps in phage isolation for therapeutic purposes are presented in **Figure 1**. Identification of new phages and adding them to the collection begins with multiplying phages obtained from an environmental sample with the use of the target bacterial host and checking the activity of the phage against strains of a well-defined bacterial collection. This procedure is known as the determination of the phage host range on a target host strain panel (Mirzaei and Nilsson, 2015; Pirnay et al., 2015). Interestingly, some phages can form plaques that are too small to be seen. An example is *Bacillus* phage G, which cannot diffuse through agar, due to its extraordinary large virion size (Hendrix, 2008).

There are many ways to improve the visibility of phage plaques on a bacterial lawn. One possible method, especially in the case of phages isolated from environmental samples, is the use of sublethal doses of antibiotics (e.g., 2.5–3.5 μg/ml of ampicillin, depending on the top agar thickness), which was suggested by Łoś et al. (2008). This allows one to obtain plaques of increased diameter or obtain any kind of visible plaques in standard conditions in the case of *E. coli* phages. Similarly, Kaur et al. (2012) also used sublethal doses of antibiotics (inhibitors of proteins synthesis) to increase the size of MR-5 phage plaques (which are usually tiny, small, and difficult to visualize) on the methicillin-resistant *S. aureus* strain. The addition to the soft agar layer of 2,3,5-triphenyltetrazolium, sodium thiosulfate or ferric ammonium citrate, as it was done in the case of *Salmonella enterica* subsp. *enterica* bacteriophages isolated from swine lagoon eﬄuent may be another way to facilitate plaque visualization (McLaughlin and Balaa, 2006).

For therapeutic purposes it is necessary to select a phage against the isolated bacterium that is the cause of infection. There may be a large number of phages in the tested sample, but only one may be identified on a particular bacterial strain lawn (Kutter, 2009).

When multiple phages are expected to be present in a tested sample, the enrichment method is used with more than one bacterium, and the obtained cell lysate may potentially be a source of many phages (Łobocka et al., 2014). In this method bacteria are usually cultivated in LB medium or in broth, sometimes in BHI broth (Beheshti Maal et al., 2015). D’Herelle (1938) recommended applying more than 50 bacterial strains in this procedure. One may even use bacteria from different taxons (genera, species) and obtain phages with a wide host range (Łobocka et al., 2014).

Liquid culture and the solid plate method are preferred for phage isolation. The double layer method is used to determine the titer of phage in the sample and/or preparation at each step of searching and preparing phage preparations used as therapeutics (Gratia, 1936; Adams, 1959). Fresh bacterial culture in the expotential phase of growth is mixed with the environmental sample sterilized by filtration (where phages are likely to be detected) and incubated properly depending on the host bacterial strain, to allow the phage to amplify and attain a high titer. If a plaque is observed on the bacterial lawn, the next step of phage isolation is to pick and transfer a single plaque into liquid medium (Mattila et al., 2015). The efficiency of plating (EOP) of some phages, e.g., the T4 phage, is 100%, which means that in optimal conditions every single particle of the phage can infect a host cell and produce plaques (Kutter, 2009). Typically, three to five passages are necessary to ensure the multiplication of a single phage from the environmental sample (Łobocka et al., 2014). The monoclonality of phage preparations is a separate issue, as commonly used phage propagation strains may be a source of contaminating undesired phages.

To detect the presence of phages in samples in which we could not detect them using the plate method, the colorimetric method is used in our laboratory (**Figure 1**). It is simple and sensitive, and it offers unequivocal results (McLaughlin, 2007; Żaczek et al., 2011). It exploits the ability of living bacterial cells to reduce the tetrazolium to formazan, which is visible as the appearance of a dark red color in the bacterial culture over time. Lysis of bacteria results in the lack of culture medium staining as compared to the control and may suggest the presence of phages in the tested samples. Advantages of this method include the possibility to detect phage particles present in the sample in a low number and the possibility to detect phage presence in many samples simultaneously during one experiment. Fischer et al. (2013) also recommended the microplate test for the rapid determination of bacteriophage susceptibility of *Campylobacter* strains. Moreover this method is suitable for automation of the phage susceptibility testing. Additionally, the microplate test allows one to examine a 5–10-fold greater number of isolated strains, compared to the conventional plate method.

A method that is useful for the acquisition of phages for cocktails with great therapeutic potential is so called step-by-step (SBS) method (Gu et al., 2012). It is based on the selection of lytic phages active against both the wild type phage-sensitive strain and its phage-resistant variant. The wild type bacteria and the first phage-resistant strain are used for isolation of the first and the second phage, and bacteria resistant to the second phage are applied to isolate the third phage.

Mirzaei and Nilsson (2015) compared the efficiency of plating (EOP) and host range of several phages using the spot test and the double-layer agar method that are commonly used in the initial phage characterization. Strains of the standard *E. coli* and *Salmonella* reference collections, and ESBL-producing *E. coli* served them to isolate phages from sewage water and waste water from Stockholm (including hospital samples). Six phages of the widest lytic spectrum were chosen for the comparative analysis of both methods. The results did not fully correlate. The authors suggested that the differences may be an effect of lysis from without, the presence of bacteriocins or endolysins in the lysate, or the presence in certain bacterial strains of prophages encoding phage resistance genes.

Most often, phage growth in laboratory conditions does not reflect its good propagation *in vivo* (Weld et al., 2004). Phages that have similar properties and may have a similar host range under laboratory conditions can have different therapeutic efficacy *in vivo*. Therefore, it is important to select for therapeutic purposes only phages that have high fitness *in vitro*, and try to simulate conditions corresponding to the site of infection to check phage potential therapeutic efficacy (Bull et al., 2010; Bull and Gill, 2014; Łobocka et al., 2014). It is believed that phage success in therapeutic application may result from appropriate phage-bacterial dynamics (Bull and Gill, 2014).

For phage screening it is recommended to use as many bacterial strains as possible. Phage typing and the host range are usually determined using a routine spot test, in which phage lysates or their dilutions are used. This method is simple, quick and not expensive, yet very useful, and it gives an overview of the bacterial sensitivity to a particular set of phages.

## Therapeutic Phages

The most common phage preparations are colloid suspensions (protein based), most often prepared freshly (Vandenheuvel et al., 2015). For therapeutic purposes bacteriophages should be dispersed in solution, stable, safe and have a high affinity to bacteria. Thus, lytic tailed phages belonging to the *Myoviridae, Siphoviridae*, and *Podoviridae* families are used for phage therapy (Wittebole et al., 2014).

The historical collection of Bacteriophages in the IIET PAS was initiated by Ludwik Hirszfeld, who was working with *Salmonella* bacteriophages. Currently, the collection contains approximately 800 phages, active against mainly such pathogens as *Escherichia, Morganella, Klebsiella, Enterobacter, Enterococcus, Pseudomonas, Staphylococcus*, and *Salmonella*, and it is being continuously expanded. In the collection there are phages that were isolated from hospital sewage, inland and marine waters, animal and human feces, water, soil, plant, and clinical materials, e.g., from patients with the diabetic foot syndrome. Our phage collection contains phages that may be active against both *K. pneumonia* PDR (resistant to carbapenems), *E. coli* O157:H7 and O104:H4 (which caused the death of more than 50 patients in Germany in 2011). The procedure of preparing phages against *S. aureus* and *P. aeruginosa* has been in our laboratory under patent protection since 2002 (EP1 406 642 B1, PL 19543781, US 7, 232564 B2).

We noticed that phages in our collection have wide lytic spectra. The ones with the widest spectrum are staphylococcal phages (mean = 95%); coliphages have mean activity against *E. coli* strains of 86% (including ESBL-positive strains); *E. faecalis* phages destroy approximately 80% of our strains; phages active against *Klebsiella* lyse 62% of our strains; whereas *P. aeruginosa* phages lyse 56%. In the case of some *Salmonella* serotypes we have phages with activity as high as 100%. We found that newly isolated bacterial strains are less sensitive to “historical” phages. This phenomenon has been observed especially in the case of *E. coli, K. pneumonia, P. aeruginosa*, and, on a lesser scale, in the case of *S. aureus* strains.

It is recommended to maintain ongoing monitoring of available phage collections and update phage cocktails with newly isolated and characterized phages, removing old and inactive ones (Gill and Young, 2011). Our laboratory’s phage collection increases constantly with phages isolated from both environmental and clinical materials. There is a possibility as well to use a preparation from our repository and quickly apply it in a patient (Fortuna et al., 2008). Even when phage sensitivity is changing during phage therapy, it is possible to find a new active phage.

Plaque morphology is important for the selection of therapeutic phages. Only clear, non-turbid plaques without a halo effect should be classified as the effect of the lytic phage activity. The obtained plaques are a complex result of different phage features, growth phase of the bacterial host, diffusion of virions in agar plate, and inactivation of phage particles by debris of bacteria (Abedon and Yin, 2009; Gill and Young, 2011). Similarities in morphology between plaques may often result from similar properties of phages that infect related bacteria. Phage adsorption from the therapeutic point of view is effective when it is fast – typically 70% or higher in 10 minutes. According to Bull and Gill (2014), it is possible to predict phage success when the phage-bacterial population dynamics is known. Successful treatment requires phages of high lytic activity – a feature that is correlated with the phage burst size (Mirzaei and Nilsson, 2015).

After phage isolation it is necessary to characterize every new phage before incorporating it into the collection. It is important to test: the latent period, the burst size (Hyman and Abedon, 2009; Mirzaei and Nilsson, 2015), host growth, “phage lifestyle”, the adsorption rate, multiplicity of infection (MOI), and stability in storage condition, morphology of plaques, phage morphology, ultrastructure, and taxonomy (Ackermann and Heldal, 2010; Gill and Hyman, 2010). Moreover, phages should be characterized by sequencing of the whole genome to exclude toxin genes, virulence factor genes, and genes responsible for lysogeny, and they should have low transducing potential (Łobocka et al., 2014). For the safety of phage application it is also necessary to remove prophages and certain other mobile genetic elements from phage propagation strains. The risk of, e.g., virulence genes’ presence in phage genomes cannot be totally excluded for safety reasons (Vandenheuvel et al., 2015). So, every single isolate should be considered as an entity with individual properties and requirements. Quality requirements for the production of phage preparation intermediate or finished products should be provided in laboratories with specified air quality and cleanliness, which minimize the risk of its contamination, and the biosafety level depends on the type of bacteria that are used in preparing formulations (Pirnay et al., 2015).

The requirement for manufacturing the BFC-1 phage cocktail for clinical trials using good manufacturing practices (GMPs) was obvious (Merabishvili et al., 2009). All products that are used for the preparation of the cocktail were certified. The preparation was cleared of endotoxins. Pirnay et al. (2015) the quality and safety requirements for sustainable phage therapy products were published. They suggested that manufacture of products for phage therapy should be described in detail at each stage of the process in standard operating procedures (SOPs) and validated.

## Criteria of Phage Selection for Therapeutic Purposes

### Phage Properties

The most important criterion for selecting phages for therapeutic application is their specificity – called affinity (Ly-Chatain, 2014). This property is of great importance for phage therapy. Therefore, one should be certain that an isolated bacterium is sensitive to the selected phage, as it may limit the effectiveness of phage application (the use of preparations such as cocktails may improve phages’ lytic spectrum) (Goodridge, 2010). To manage the problem associated with phage specificity it is advisable to use phage cocktails which have broader lytic activity against bacteria than single phages. Phages with broad spectrum-strain lytic activity should be selected. Phage specificity for certain species of Gram-positive and Gram-negative bacteria (including antibiotic-resistant strains, e.g., MRSA, VRE, ESBL-producing *E. coli*, and multidrug resistant *P. aeruginosa*) allows one to eliminate pathogenic bacteria without affecting the natural microflora. There are suggestions that some phages show family rather than genus or species specificity (Gill and Hyman, 2010).

There are numerous criteria that allow us to classify phages as useful for therapeutic purposes, including efficacy of phage therapy and the possibility to avoid adverse effects (Denou et al., 2009). First of all it is postulated to use only lytic phages. Temperate phages often give visibly turbid plaques (Gill and Young, 2011). According to Ackermann (2005), it is estimated that approximately half of the phages isolated from the environment are temperate. It is important for consideration, because most therapeutic phages are isolated from environmental samples. The presence of prophages within a bacterial strain may cause lysogenic conversion, which undermines phage applicability in therapy. Therefore, phages that contain genes for lytic phage repressors, integrases or transposases should not be used for therapeutic purposes (Łobocka et al., 2014). It is especially important, because certain mutants of temperate phages may cause the formation of clear plaques (Weigle, 1953).

For safety reasons, every phage that has potential for therapeutic application should be sequenced. Especially the possibility of phage genome participation in horizontal gene transfer should be investigated (Pirnay et al., 2015; Davies et al., 2016). Phages have not been demonstrated to have adverse effects on eukaryotic cells, and they should be safe for humans and animals.

### Phage Stability under Storage Conditions and Formulations

Phages are composed of protein structures, and therefore they may be instable in solution form (Vandenheuvel et al., 2015). It is recommended to adjust the type of storage of phages to their biology and properties. Most often, the best way of their storage is cooling. Sometimes it is possible to add substances that may enhance phage stability in water suspension, or to preserve by freeze-drying, spray drying, or encapsulation. Some phages are considered as stable when their titer does not significantly decrease for a few days, while others preserve their stability for years. In the case of preparations applied for therapeutic purposes, there are quality and safety requirements that storage conditions should be validated and monitored (Pirnay et al., 2015).

Phage storage should assure the stability of phage particles in the form and conditions in which the preparation is stored, but the form of application should also protect phage particles against losing their activity. For example, Cooper et al. (2014) tested a cocktail of three phages (phages GL-1, GL-1_2.5_, and LP-M_10_) active against *P. aeruginosa* in the form intended for nebulized formulation. The assessed phages retained their activity over 180 days at a storage temperature of 4°C as well as at room temperature, and they were successfully nebulized with little loss of infectivity. However, the endotoxin levels [as verified by the Limulus amoebocyte lysate (LAL) test] were considerably higher than the acceptable levels.

Phage particles’ stability in a preparation intended to be used in humans may depend on the composition of the phage preparation. O’Flaherty et al. (2005) observed that the K phage active against *S. aureus* showed stability in bismuth-based cream and this cream exhibited bactericidal activity also several days after storage at room temperature. What is more, in a semi-solid preparation – a Cetomacrogol cream – at a concentration of 2.5 × 10^8^ pfu/ml per gram, *Propionibacterium acnes* phages remained active for 90 days when the preparation was stored at 4°C in a light-protected place (Brown et al., 2016). A non-ionic cream base preparation was chosen because it excludes potential occurrence of interactions of ions with phage particles. These properties, especially stability and its easy-to-use form, assure this preparation’s potential for topical treatment of acne infection. Esteban et al. (2014) stabilized the K-phage and delivered it via an oil-in-water nanoemulsion. Freshly prepared K/nanoemulsion exhibited greater bactericidal properties than freely suspended bacteriophage. The emulsions containing the K phage killed cells of three different strains of *S. aureus.* Similar observations were obtained for preparations that were stored at room temperature and at 4°C, for up to 10 days. It was observed that bacteriophage activity was enhanced when it was delivered via nanoemulsions. Tablets and powders containing phages may ensure the phage titer and its stability in low humidity conditions (Schade and Caroline, 1944c).

The shelf life of phage preparations should be monitored, as phages differ in their titer’s stability under storage conditions, and its persistence depends on suspended media as well as on different physical and chemical external conditions (Jończyk et al., 2011). A good candidate for phage therapy is a phage that can easily propagate in both laboratory and industrial conditions and is characterized by stability in storage conditions. For some phages storage in lysate form at 4°C is required, as no significant loss of its titer is observed (Ackermann et al., 2004). For other phages better conditions that allow titer stability to be maintained may be freezing at -80°C with the addition of glycerol or freeze-drying. Golec et al. (2011) offered a reliable method of preservation of tailed phages. It involves storage of phages in the DNA form frozen inside the infected bacterial cells at -80°C without observing any significant decrease in phage and host viability. The authors suggested that this method may be useful for newly isolated phages that have an unknown structure and biology and whose propagation conditions have not been determined and optimized. They also recommend this method as suitable for phage preservation. Puapermpoonsiri et al. (2010) indicated that the secondary drying cycle following lyophilization is most important for maintaining phage stability. Phages active against *Pseudomonas aeruginosa* and *S. aureus* were encapsulated into biodegradable microspheres (for inhalation) based on emulsification and freeze-drying, and it was observed that only a partial loss of phage lytic activity took place, which may result from the exposure of the phage particles to the water-dichloromethane interface, with the lyophilization process itself having little effect (Puapermpoonsiri et al., 2009). Also, Alfahdel et al. (2011) used *S. aureus* phage lyophilization in a viscous solution of 1–2% (w/v) hydroxypropyl methylcellulose (HPMC) with/without the addition of 1% (w/v) mannitol, yielding nasal inserts composed of a highly porous leaflet-like matrix. Phages were homogeneously distributed in the dried matrix. Their titers decreased 10-fold following lyophilization to 10^8^ pfu per insert; and further 100- to 1000-fold loss of pfu was observed during 6–12 months of storage at 4°C. It is possible to add substances, e.g., albumin, salts, or gelatin, to improve stability; but in the case of phages in pharmaceutical application it may be problematic (Gill and Hyman, 2010). Tovkach et al. (2012) prepared an *Erwiniophage* ZF40 suspension with the addition of a magnesium and gelatin STMF buffer which ensured the stability in long-term preservation of unstable enterobacterial phages (+4 to -2°C). The authors mentioned that the possible problems with long-term storage may be the result of phage virions’ or DNA structural instability. Schade and Caroline (1944b,c) tested the influence of lyophilization on phage stability. They observed that that addition of, for example, yeast extract, lecithin, and raw egg white, protected dysentery phage Sh-1, promoting the stability of this sensitive phage during lyophilization. Interestingly, the dysentery phage was protected against damage that could be the result of lyophilization, and obtained powders were stable during 12 months of storage over a desiccant at 37°C (Schade and Caroline, 1944a). Also phages active against *S. aureus*, including MRSA strains, were lyophilized with the presence of different stabilizers: sucrose, trehalose, mannitol, and glycine, polyvinylpyrrolidone and PEG 6000 (polyethylene glycol) at different concentrations (Merabishvili et al., 2013). Trehalose and sucrose (0.5 M) were found to be the best additives protecting the ISP phage, whose titer dropped by 1 log after lyophilization and by another one after being stored in powder form for 37 months at 4°C.

Fortier and Moineau (2009) stated that lyophilization is a more effective method to preserve phage stability than storage in a solution, and lyophilized phages had higher heat stability and were resistant to drying. As they observed, phage protective action was provided in egg yolk. Other researchers observed that storing the BFC-1 phage cocktail for 12 months at 4°C, did not cause the observed loss of stability (Merabishvili et al., 2009).

Importantly, inactivation of phage particles may occur before reaching the site of infection. This situation should be possible to avoid; therefore the initial dose and the route of phage administration should enable them to circulate, achieve the infection site and multiply there as long as pathogenic bacteria are present (Gill and Young, 2011). However, suspensions of phages that exceed 10^13^ pfu/ml seem to be difficult to obtain since concentrated suspensions become too viscous (Nilsson, 2014).

Phage preparations that are used as therapeutics may contain only one phage or be a cocktail consisting of two or more phages. The latter type of preparation’s composition may prevent the occurrence of cross-resistance, and, based on this phenomenon, a bacterium which is resistant to one phage may remain sensitive to another, and cocktails that contain phages using different receptors for binding to bacteria may be a better solution for eliminating the development of resistance in bacteria (Gill and Hyman, 2010). Some changes in lytic patterns of patients’ strains isolated during the course of phage therapy may be observed. It may be due to instability and heterogeneity of bacterial strains as well as different bio- and serotypes of bacteria or changing phage receptors. According to Chan and Abedon (2012), the treatment of infections caused by antibiotic-resistant bacteria seems to be more effective when a phage cocktail is used, compared with single phage efficacy.

## Limitations of Phage Procurement for Therapeutic Purposes

The most important factor for effective use of phages as therapeutics is the widest possible knowledge (Goodridge, 2010) regarding biology of phages, their individual properties and stability in different forms of available preparations (both lysates and purified preparations). There are opinions that phage treatment may induce the production of antiphage antibodies and this may limit the phage antibacterial activity (Sulakvelidze et al., 2001). Moreover, it is possible that bacteria may develop resistance to phages. The next limitation is the lack of legislative solutions adapted to broad use of bacteriophages to combat antibiotic-resistant bacteria.

The presence of phages of a wide host range in the collections, on one hand, allows the number of phages needed in a collection to be reduced, and on the other hand, it offers the opportunity to increase the probability of finding phages active against emerging drug-resistant bacterial strains (Mirzaei and Nilsson, 2015). The phage searching and isolation procedure should be designed in such a way as to prevent possible treatment failure resulting from wrong phage selection (Gill and Hyman, 2010).

Clear lysis zones observed on a bacterial lawn are most often the result of phages’ lytic activity. Sometimes, it is possible for a phage preparation to contain a factor that causes lysis but it is not directly connected with phages’ lytic activity (Hyman and Abedon, 2010). The agents responsible for the observed effect could be bacteriocins, endolysins (that may cause lysis from without). Additionally, abortive infection can cause bacterial death without the release of new phage progeny. Interestingly, filamentous phages form plaques, but they do not lead to the lysis of bacterial cells (Gill and Hyman, 2010). Also *Bdellovibrio* bacteria can form plaques (Hobley et al., 2006).

It is extremely important to keep in mind that there are differences between phages themselves, and the type of their isolation, tested material, and thus the type of procedure during phage propagation should be adjusted to an individual phage and should depend on the type of host bacteria in which phages are propagated (Mattila et al., 2015). Sometimes it is not possible to detect any plaques on the bacterial lawn. The reason for this phenomenon may be that the bacterial host is insensitive to the phage, and it does not indicate that there is no phage in the sample but that the wrong bacteria were chosen (Gill and Young, 2011) or that phage particles could disintegrate (Gill and Hyman, 2010). One of the isolation limitations may result from the differences in propagation ability of phages under experimental conditions or from the inability to isolate phages which are present in the sample (Hendrix, 2002). Bacteria that are present in environmental samples are unculturable (we cannot isolate them, or we do not know the culture requirements) in laboratory conditions with commonly available equipment and methods, and therefore we are able to isolate only a small number of phages occurring in these sources. Cultures used in the search for phages occurring in water samples should be incubated at 25°C rather than 37°C. Interestingly, it is of great importance to adjust proper culture conditions to a particular bacterial host’s requirements. Procurement of phages generally may seem to be easy, but matching them for therapeutic purposes should be adjusted to individual phage properties. Other limitations may be due to a large number of genetic changes in bacterial and phage strains. This phenomenon may be in part a result of the acquisition of resistance to phages by bacteria.

Phage virions within the mammalian body are exposed to factors that may cause their inactivation. Contact with the acidic gastric pH (Verthé et al., 2004), alkaline bile salts (Ma et al., 2008), antibodies (Keller and Traub, 1974) and phagocytes, as well as the toxicity of urea, may result in the loss of their lytic activity. In order to avoid adverse factors, searching for improved methods of administration and formulations for new forms that reduce the exposure of phage to destructive conditions is desirable. For example, the encapsulation of phages may enable phage particles to retain the activity in unfavorable conditions (Dini et al., 2012; Colom et al., 2015).

Therapeutic phages are currently applied in the form of lysates or purified preparations, and they cannot be toxic (Skurnik and Strauch, 2006; Skurnik et al., 2007). Due to the necessity to keep the final preparations safe, it is essential that the media used for phage cultivation and propagation are definitely free from such dangerous contaminants as prions, viruses and allergens (Goodridge and Abedon, 2008).

In order to prevent an anaphylactic reaction in response to bacterial components, such as endotoxins (which may be a component of the phage lysate), some investigators recommend the use of purified preparations (Skurnik and Strauch, 2006; Loc-Carrillo and Abedon, 2011). However, while it is thought that the staphylococcal phage lysates stimulate the body’s immune response, purified preparations may show immunosuppressive activity (Górski et al., 2012). Purification of phage preparations of bacterial endotoxins is becoming more effective. Endotoxin levels in preparations for intravenous use should not exceed 5 endotoxin units (EU 1 ≈ 100 pg) per kilogram of body weight per hour (Wanninger and Artz, 2009). The most common method uses isopycnic density gradient centrifugation. Sometimes this method may be problematic – phage particles may be damaged by high salt concentrations (Gill and Young, 2011). There are methods, e.g., ion exchange chromatography or affinity chromatography (Oślizło et al., 2011; Narasimhaiah et al., 2013), which allow one to obtain purified preparations with reduced endotoxin content. Other methods used for phage purification may be cesium chloride (CsCl) gradient centrifugation or sucrose gradient (Kleiner et al., 2015). The yield of phages using the CsCl method is low, and some phages may be lost due to aggregation or damage of virions. High performance liquid chromatography (HPLC) may also be applied successfully to concentrate and purify tailed phages (Adriaenssens et al., 2012). Both the methods of staphylococcal phage purification (P391328) and the method of obtaining bacteriophage preparations containing trace amounts of endotoxins (P382800; US 20100227376 A1) have been used in our laboratory under patent protection. For phage purification, Merabishvili et al. (2009) recommended the use of the Endotrap blue chromatographic column that binds endotoxins.

## Modified Phages

Apart from isolating naturally occurring phages from environmental samples, it is possible to obtain genetically modified phages (by recombination of phage genomes, site-directed mutagenesis, selection of spontaneous mutants or phage display methods). These methods offer a great therapeutic tool for dealing with the challenges of phage therapy (Moradpour and Ghasemian, 2011; Chhibber and Kumari, 2012; Dąbrowska et al., 2014). Modified phages may be deplated of undesired properties, may have changed specificity or improved therapeutic potential, as e.g., phages that have dual activity – antitumor and bactericidal – without an observable reduction in antimicrobial activity (Dąbrowska et al., 2014). These findings open new possibilities for the use of phage therapy.

The discovery of bacterial adaptive immunity system, CRISPR/Cas (Clustered Regularly Interspaced Palindromic Repeats [CRISPR]/CRISPR-associated [Cas]) systems, has opened new possibilities in the design of a genetic load that can be delivered by bacteriophages. In nature, bacteria can acquire resistance to a bacteriophage by the incorporation of the genome fragment of this bacteriophage as a spacer between repeats in the CRISPR locus (Szczepankowska, 2012). In the case of the CRISPR/Cas systems of type II, small CRISPR RNAs (crRNAs) produced by the processing of CRISPR locus transcripts serve to guide a nucleolytic enzyme to target invading phage or other DNA molecules, if they are only complementary to them. The CRISPR/Cas systems were also found in some phages (Hargreaves et al., 2014; Zheng et al., 2016). A CRISPR/Cas system that is encoded by a virulent *V. cholerae* phage is used to destroy the DNA of its host chromosomal island, which excises from the chromosome in response to phage infection, circularizes and interferes with phage development (Seed et al., 2013). This natural system provides a proof of the concept that phage-mediated delivery of CRISPR/Cas-encoded RNA-guided nucleases (RGNs) is an efficient way to target bacterial DNA in a sequence specific manner. Indeed staphylococcal ϕMN1phage particles packed with engineered phagemid DNA carrying CRISPR/Cas with spacers targeting certain *S. aureus* virulence genes killed virulent, but not avirulent *S. aureus* (Bikard et al., 2014). They could also reduce the number of virulent *S. aureus* strain cells on a skin of infected mice. Modified phages with multiple varying CRISPR spacers were effective in destruction of multiple DNA targets in the infected bacterial cell.

Phages of changed properties may be also obtained by chemical modifications. Modifications of phage particles by attaching monomethoxy-polyethylene glycol (mPEG) to proteins caused the phage to become less immunogenic. The PEGylated phage caused a decrease in the level of cytokines such as IFN-γ and IL-6 in both the non-immunized and the phage immunized mice (Kim et al., 2008). The modified phage particles were characterized by a longer half-life in the organism, which may be of practical importance in enhancing the effectiveness of phage therapy. Our recent hypothesis suggests that it might be possible to obtain modified phages with homing peptides that enable phage localization in infected tissues (Górski et al., 2015). Taken together, all kinds of phage modifications open new possibilities for the use phages in antibacterial therapies.

## Concluding Remarks

Phage therapy offers a real chance for patients suffering from infections caused by antibiotic-resistant bacteria. Widening our knowledge about phage biology, their individual properties and stability in different forms of available preparations offers the most significant prospect for effective application of phages as therapeutics (Goodridge, 2010).

Phage searching and isolation procedures should be adjusted to the type of bacterial strain being the host of the phage, which would also prevent treatment failures that may lead to phage misselection. Phage preparations intended for treating infections in humans should be safe, sterile and endotoxin-free. What is more, the methods of administration and formulations for new forms that reduce the exposure of phages to destructive conditions should be improved. Genetically modified phages with broader activity may be obtained, and it is possible to shape their activity depending on the therapeutic needs. The progress in the knowledge about phage genomics, immunobiology and experimental therapy in animals and in humans suggest that phages could become the antibacterial drugs of the 21st century.

## Author Contributions

BW-D and EJ-M drafted the main part of the manuscript. MŻ performed part of manuscript and the **Figure 1**, MŁ and MŁ-S prepared parts of manuscript. AG gave support and conceptual advice at all stages of manuscript preparation. All authors revised the manuscript.

## Conflict of Interest Statement

The authors declare that the research was conducted in the absence of any commercial or financial relationships that could be construed as a potential conflict of interest.

BW-D and AG are inventors of a patent (EP1 406 642 B1, PL 19543781, US 7, 232564 B2) for procedure of preparing phages against *S. aureus* and *P. aeruginosa* (P382800; US 20100227376 A1) and a method of obtaining bacteriophage preparations containing trace amounts of endotoxins owned by the Institute of Immunology and Experimental Therapy. EJ-M, MŁ, MŻ, MŁ-S, declares no potential conflict of interest.
