# Corrigendum: Bacteriophage Procurement for Therapeutic Purposes

**DOI:** 10.3389/fmicb.2016.01813

**Published:** 2016-11-09

**Authors:** Beata Weber-Dąbrowska, Ewa Jończyk-Matysiak, Maciej Żaczek, Małgorzata B. Łobocka, Marzanna Łusiak-Szelachowska, Andrzej Górski

**Affiliations:** ^1^Bacteriophage Laboratory, Ludwik Hirszfeld Institute of Immunology and Experimental Therapy, Polish Academy of SciencesWroclaw, Poland; ^2^Phage Therapy Unit, Ludwik Hirszfeld Institute of Immunology and Experimental Therapy, Polish Academy of SciencesWroclaw, Poland; ^3^Institute of Biochemistry and Biophysics, Polish Academy of SciencesWarsaw, Poland; ^4^Autonomous Department of Microbial Biology, Faculty of Agriculture and Biology, Warsaw University of Life SciencesWarsaw, Poland; ^5^Department of Clinical Immunology, Transplantation Institute, Medical University of WarsawWarsaw, Poland

**Keywords:** bacteriophage isolation, therapeutic phages, experimental phage therapy, treatment of bacterial infections, antibiotic resistance

In the original article, we neglected to include the Wrocław Centre of Biotechnology as a funding source. The full Funding statement should read as follows:

This work was financially supported by the project “Innovative Bacteriophage Preparation for the Treatment of Diabetic Foot” no. POIG.01.03.01-02-048/12 funded by the National Center for Research and Development. MŁ contribution was financially supported by the statutory funds for the Institute of Biochemistry and Biophysics, PAS. Publication was also supported by Wrocław Centre of Biotechnology, programme, the Leading National Research Centre (KNOW) for years 2014–2018.

The authors apologize for this oversight. This error does not change the scientific conclusions of the article in any way.

## Conflict of interest statement

BW-D and AG are inventors of a patent (EP1 406 642 B1, PL 19543781, US 7, 232564 B2) for procedure of preparing phages against *S. aureus* and *P. aeruginosa* (P382800; US 20100227376 A1) and a method of obtaining bacteriophage preparations containing trace amounts of endotoxins owned by the Institute of Immunology and Experimental Therapy. EJ-M, MŁ, MŻ, MŁ-S, declares that the research was conducted in the absence of any commercial or financial relationships that could be construed as a potential conflict of interest.

